# Astragalus polysaccharide ameliorates insulin resistance in HepG2 cells through activating the STAT5/IGF‐1 pathway

**DOI:** 10.1002/iid3.1071

**Published:** 2023-11-22

**Authors:** Xinxin Yue, Wei Hao, Min Wang, Yang Fu

**Affiliations:** ^1^ Department of Clinical College HE University Shenyang Liaoning China; ^2^ Department of Burn and Plastic Surgery General Hospital of Northern Theater Command Shenyang Liaoning China

**Keywords:** astragalus polysaccharide, HepG2 cells, insulin resistance, the STAT5/IGF‐1 pathway

## Abstract

**Background:**

Insulin resistance (IR) is considered as a major factor initiating type 2 diabetes mellitus and can lead to a reduction in glucose uptake that mainly occurs in the liver. Astragalus polysaccharide (APC), extracted from the traditional Chinese medicine, has been recorded to suppress IR. However, the underlying mechanism remains inadequately explored.

**Methods:**

IR was induced in HepG2 cells which further underwent APC treatment. Cell viability was determined by cell counting kit‐8 assay. Pretreatment with AG490, an inhibitor of signal transducer and activator of transcription 5 (STAT5) signaling, was performed for investigating the influence of STAT5 on APC. Glucose uptake level was reflected by 2‐deoxyglucose‐6‐phosphate content determined through colorimetric assay. Expression levels of insulin‐like growth factor 1 (IGF‐1), IGF‐1 receptor (IGF‐1R), phosphorylated‐STAT5/STAT5, and p‐protein kinase B (AKT)/AKT in the cells were assessed by Western blot. Radioimmunoassay (RIA) was used to detect IGF‐1 secretion in the cells.

**Results:**

APC at doses of 10 and 20 mg increased the viability of HepG2 cells with/without IR induction, and abrogated IR‐induced inhibition of glucose intake. Meanwhile, APC (10 mg) offset IR‐induced inhibition on the expressions of IGF‐1R and IGF‐1, the activation of AKT and STAT5, and the secretion of IGF‐1 in HepG2 cells. More importantly, the reversal effect of APC on IR‐induced alterations in HepG2 cells was counteracted by AG490.

**Conclusion:**

APC ameliorates IR in HepG2 cells through activating the STAT5/IGF‐1 pathway.

## 1 INTRODUCTION

Insulin resistance (IR) refers to a reduction in insulin‐stimulated glucose uptake and utilization by diverse tissues such as the liver, muscles, and fats.^1^ While genetic factors are determinants, the risk of developing IR is highly associated with obesity.^2^ The intake of massive saturated fatty acids, the main component of fats, results in disordered metabolism of glucose and lipid in the body,^3^ and further incurs the release of inflammatory factors,^4^ which can affect the activation of the insulin signaling pathway, thereby inducing IR.^5^, ^6^ IR is the main factor initiating type 2 diabetes mellitus (T2DM), the most prevalent chronic noncommunicable disease in the world,^1^ as IR individuals cannot secrete sufficient insulin to overcome IR‐caused attenuation in glucose disposal. Therefore, delving into the molecular mechanisms underlying IR is important to the development of interventions in T2DM.

The liver is a major organ for storing and metabolizing glucose and producing endogenous glucose, which thus usually appears as the first site where IR occurs.^7^ Following IR, various glucose metabolism‐associated enzymes become dysregulated, leading to increased blood glucose levels.^8^ Insulin‐like growth factor 1 (IGF‐1), also known as somatomedin C, is a protein hormone similar to insulin in molecular structure, and possesses autocrine, paracrine, and endocrine properties.^9^ IGF‐1 is mainly generated from the liver in response to growth hormone (GH) stimulation.^10^, ^11^ A lower level of IGF‐1 can be caused by liver disease and uncontrolled diabetes mellitus^12^ and is linked to IR.^13^ IGF‐1 plays a crucial role in fetal development, childhood growth, muscle regeneration as well as homeostasis maintenance in adult tissues by regulating cell survival, proliferation, and differentiation.^12^ The function of IGF‐1 is mediated mostly by insulin‐like growth factor type 1 receptor (IGF‐1R), a tyrosine kinase which is required in cell growth, cell development and cell cycle maintenance, and can potently activate the protein kinase B (AKT) signaling that is involved in glucose transporter 4‐mediated glucose uptake.^13^, ^14^, ^15^ IGF‐1 controls the homeostasis of glucose through a direct action on the balance between GH and insulin.^16^ More importantly, Q. Li et al.'s^17^ study has shown that IGF‐1 upregulation contributes to suppression of IR, indicating a regulatory role of IGF‐1 in IR.

Signal transducer and activator of transcription 5 (STAT5) is a transcription factor that mediates signaling pathways activated by GH.^18^ Upon GH stimulation, STAT5 is activated, which further controls the transcription of IGF‐1 and modulates gene expression to reduce lipid synthesis and/or storage.^19^, ^20^ Also, STAT5 is required for IGF1 upregulation to combat IR and hepatic cholesterol accumulation.^17^

Furthermore, a research has revealed that STAT5a expression is upregulated in rats with experimental colitis during treatment with astragalus polysaccharide (APC).^21^ APC is an important bioactive ingredient derived from Huangqi (*Radix Astragali Mongolici*), a Chinese herb whose origin can be traced back to more than 2000 years ago.^22^ By virtue of antioxidative, antihypertensive, antitumor, immunomodulatory and antiviral properties, APC has been worldwide recognized as a potent drug for the treatment of physiological conditions and diseases.^23^, ^24^ Notably, APC can be applied to treat T2DM.^25^ Previous studies have reported that APC can serve as a candidate for promoting insulin sensitivity of the adipocytes, the muscle and the liver.^25^, ^26^, ^27^ However, whether APC modulates the STAT5/IGF‐1 pathway to regulate hepatic IR remains unexplored.

In our study, we probed into the regulatory role of APC in hepatic insulin sensitivity, and explored whether and how the activation of the STAT5/IGF‐1 pathway participates in the regulation of APC.

## 2 METHODS AND MATERIALS

### 2.1 Culture of HepG2 cells

HepG2 cells were purchased from Procell Life Science & Technology (CL‐0103), and maintained in Minimum Essential media (MEM; PM150410; Procell Life Science & Technology) supplemented with 5% fetal bovine serum (FBS; 164210‐500; Procell Life Science & Technology) and 1% Penicillin‐Streptomycin (PB180120; Procell Life Science & Technology) at 37°C with 5% CO_2_ in a humid atmosphere.

### 2.2 IR induction

IR was induced in HepG2 cells as per previous description.^28^ Briefly, HepG2 cells were starved in MEM containing 0.5% FBS for 12 h, and then treated for 18 h with the glucosamine (S1635; Beyotime) whose concentration had been adjusted to 18 mM in a serum‐free MEM. Thereafter, the cells were stimulated with 100 nM insulin (HY‐P0035; MedChemExpress) for 30 min. Western blot was conducted to measure AKT signaling for validating whether the HepG2/IR cell model was successfully established by IR induction.

### 2.3 APC treatment, AG490 pretreatment, and grouping

APC (B20562, 98% purity), with the batch number of C11M7Y10255, was provided by Shanghai Yuan Ye Biotechnology, which was mainly composed of 75.19% glucose as well as a small amount of fructose, galactose, arabinose, and xylose. HepG2 cells and HepG2/IR cells were both treated for 24 h with APC that had been dissolved in MEM and then prepared into different doses of 0, 5, 10, 20, and 40 mg.^29^

To investigate the role of STAT5 activation in APC treatment against IR, AG490 (658411, 10 µg/mL, an inhibitor of the Janus kinase [JAK]/STAT signaling pathway; Sigma‐Aldrich), and dimethyl sulphoxide (DMSO; 472301; Sigma‐Aldrich) that served as the vehicle for AG490 were employed to treat HepG2 cells for 30 min.^17^

For grouping, cells were distributed into five groups: Control group (HepG2 cells), IR group (HepG2/IR cells), IR + APC^−^10 group (HepG2/IR cells were treated with 10 mg AP for 24 h), IR + APC + DMSO group (following DMSO pretreatment for 30 min, HepG2 cells were subjected to IR induction and then treated with 10 mg APC for 24 h), and IR + APC + AG490 group (after AG490 pretreatment for 30 min, HepG2 cells were subjected to IR induction and then treated with 10 mg APC for 24 h).

### 2.4 Cell counting kit (CCK)‐8 assay

HepG2 cells and HepG2/IR cells were separately placed in 96‐well plates at a density of 5 × 10^3^ cells/well, and then treated with different doses (0, 5, 10, 20, and 40 mg) of APC for 24 h. Later, CCK‐8 reagent (C0037; Beyotime) was added into the cells at a ratio of 1:10, followed by 2‐h incubation at 37°C. Cell viability was then determined at a wavelength of 450 nm via a microplate reader (GM2010; Promega).

### 2.5 Glucose uptake measurement

2‐deoxyglucose‐6‐phosphate (2‐DG6P) content was determined to reflect glucose uptake in HepG2 cells using a Glucose Uptake Colorimetric Assay Kit (K676‐100; BioVision) according to the manufacturer's protocols. Briefly, HepG2 cells and HepG2/IR cells pretreated with AG490/DMSO or not were separately seeded in 96‐well plates (1.5 × 10^3^ cells/well), and then the HepG2/IR cells were additionally subjected to 24‐h treatment in the presence or absence of APC (10 mg). Subsequently, the cells were washed twice with phosphate‐buffered saline (PBS; 806552; Sigma‐Aldrich), and starved overnight in a serum‐free MEM to achieve a more obvious degree of glucose uptake. After being washed with PBS, the cells were again starved for glucose through incubation with Krebs‐Ringer‐Phosphate‐HEPES (KRPH) buffer (MG6617; MesGen Biotechnology) containing 2% Bovine Serum Albumin (A1933; Sigma‐Aldrich) for 40 min. Later, the cells were stimulated by 1 µM insulin for 20 min to activate glucose transporter, and were subsequently incubated with 2‐deoxyglucose (10 µL, 10 mM) for 20 min. For biochemical reaction, the cells were first reacted with Reaction Mix A for 1 h at 37°C for nicotinamide adenine dinucleotide phosphate (NAPDH) generation. Second, unused nicotinamide adenine dinucleotide phosphate (NADP) was initially heated with extraction buffer and then added with neutralization buffer after cooling to complete degradation. Third, Reaction Mix B was applied to initiate enzymatic recycling amplification reaction. Lastly, the absorbance (412 nm) was measured via a microplate reader (GM2010; Promega).

### 2.6 Western blot

Whole‐cell proteins from HepG2 cells in the five groups mentioned above were extracted with Radio‐Immunoprecipitation Assay Buffer (89901; Thermo Fisher) containing Halt Protease and Phosphatase Inhibitor Cocktail (78446; Thermo Fisher). Subsequent to the determination of the protein concentration using bicinchoninic acid kit (A53227; Thermo Fisher), the proteins (45 μg) were separated on 8%–12% SDS‐PAGE gel (P0678, P0670, and P0672; Beyotime). Thereafter, the separated proteins were transferred onto polyvinylidene fluoride membranes (P2438; Sigma‐Aldrich), followed by being blocked by 5% defatted milk at room temperature for 1 h, and washed with Tris Buffered Saline with 1% Tween 20 (TBST; TA‐125‐TT; Thermo Fisher). Afterward, the blocked membranes were incubated at 4°C overnight with primary antibodies against phosphorylated (p)‐STAT5 (ab32364, 90 kDa, 1:1000; Abcam), STAT5 (ab230670, 90 kDa, 1:500; Abcam), IGF‐1 (ab134140, 22 kDa, 1:20,000; Abcam), IGF‐1R (ab182408, 156 kDa, 1:1000; Abcam), p‐AKT (ab38449, 56 kDa, 1:1000; Abcam), AKT (ab18785, 56 kDa, 2 µg/mL; Abcam), and glyceraldehyde 3‐phosphate dehydrogenase (GAPDH; mouse, ab8245, 37 kDa, 1:10,000; Abcam). After washing thrice with TBST, the membranes were incubated with secondary antibody, goat anti‐rabbit IgG (31460, 1:10000; Thermo Fisher) or goat anti‐mouse IgG (#G‐21040, 1:10,000; Thermo Fisher). Later, immunoreactive signals were detected with the enhanced chemiluminescence reagent kit (WP20005; Thermo Fisher) and quantified using an imaging system (Tanon 5200).

### 2.7 Radioimmunoassay (RIA)

Alterations in IGF‐1 secretion were detected by a highly sensitive RIA kit provided by Beijing Sino‐UK Institute of Biological Technology (HY‐10119). In short,^17^ HepG2 cells in each group were harvested and centrifuged at 2000*g* for 20 min, after which cell supernatant was obtained. Thereafter, dilution of standard solution was conducted by buffer solution A into six bottles of solution with gradient concentrations of IGF‐1. Following this, the diluted standard solutions (100 µL) and samples (100 µL) were separately placed and mixed with both I^125^‐labeled IGF‐1 (100 µL) and secondary antibody (100 µL) for further 24 h of incubation at 4°C. Later, the incubated solution was precipitated for 30 min and centrifuged at 3000*g* for 20 min to obtain the precipitate. Finally, the gamma count was determined by an RIA gamma counter (WIZARD2, 2470‐0150; PerkinElmer).

### 2.8 Statistical analysis

All statistical analyses were performed by Graphpad prism (version 8.0; GraphPad Software Inc.). Each result was obtained from an experiment performed in triplicate, and measurement data were expressed as mean ± standard deviation (SD). Comparisons among multiple groups were analyzed by one‐way analysis of variance (ANOVA). *p* < .05 was considered to be statistically significant.

## 3 RESULTS

### 3.1 APC at doses of 10 and 20 mg increased the viability of HepG2 cells with/without induction of IR, and abrogated IR‐induced inhibition of glucose intake

**Figure 1:**
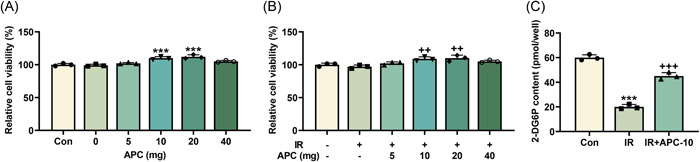
APC at doses of 10 and 20 mg increased the viability of HepG2 cells and HepG2/IR cells, and offset IR‐related inhibition of glucose intake. (A and B) The viability of HepG2 cells which were subjected to IR induction (B) or not (A) and then treated with different doses (0, 5, 10, 20, and 40 mg) of APC was determined by CCK‐8 assay. (C) Glucose uptake level in HepG2 cells which were subjected to IR induction and then treated with 10 mg APC was reflected by 2‐DG6P content determined through colorimetric assay. ***p or ^+++^ *p* < .001; * versus Con; ^+^ versus IR; ^++^ *p* < .01. 2‐DG6P, 2‐deoxyglucose‐6‐phosphate; APC, astragalus polysaccharide; CCK‐8, cell counting kit‐8; Con, control group; IR, insulin resistance.

APC (0, 5, 10, 20, and 40 mg) treatment was performed in HepG2 cells and in IR‐induced HepG2 cells. The results of CCK‐8 assay, as presented in Figure 1A,B, revealed that treatment with APC at doses of 10 and 20 mg resulted in an increase of the viability of both HepG2 cells (*p* < .001) and HepG2/IR cells (*p* < .01). Strikingly, the optimal dosages of APC for cell survival were 10 and 20 mg. Given that 10 mg of APC treatment was potent enough to restore the viability of HepG2/IR cells, it was chosen for the following experiments to investigate the pharmacological actions of APC. Then, to assess glucose intake, the content of 2‐DG6P, a glucose intake metabolite that cannot be further metabolized, was determined. It turned out that HepG2/IR cells exhibited a decreased content of 2‐DG6P as compared with the control cells (*p* < .001, Figure 1C). Moreover, APC treatment increased the content of 2‐DG6P in HepG2/IR cells (*p* < .001, Figure 1C).

### 3.2 APC offset IR‐induced effects on the expression levels of IGF‐1R and IGF‐1, and the activation of AKT and STAT5 in HepG2 cells

**Figure 2:**
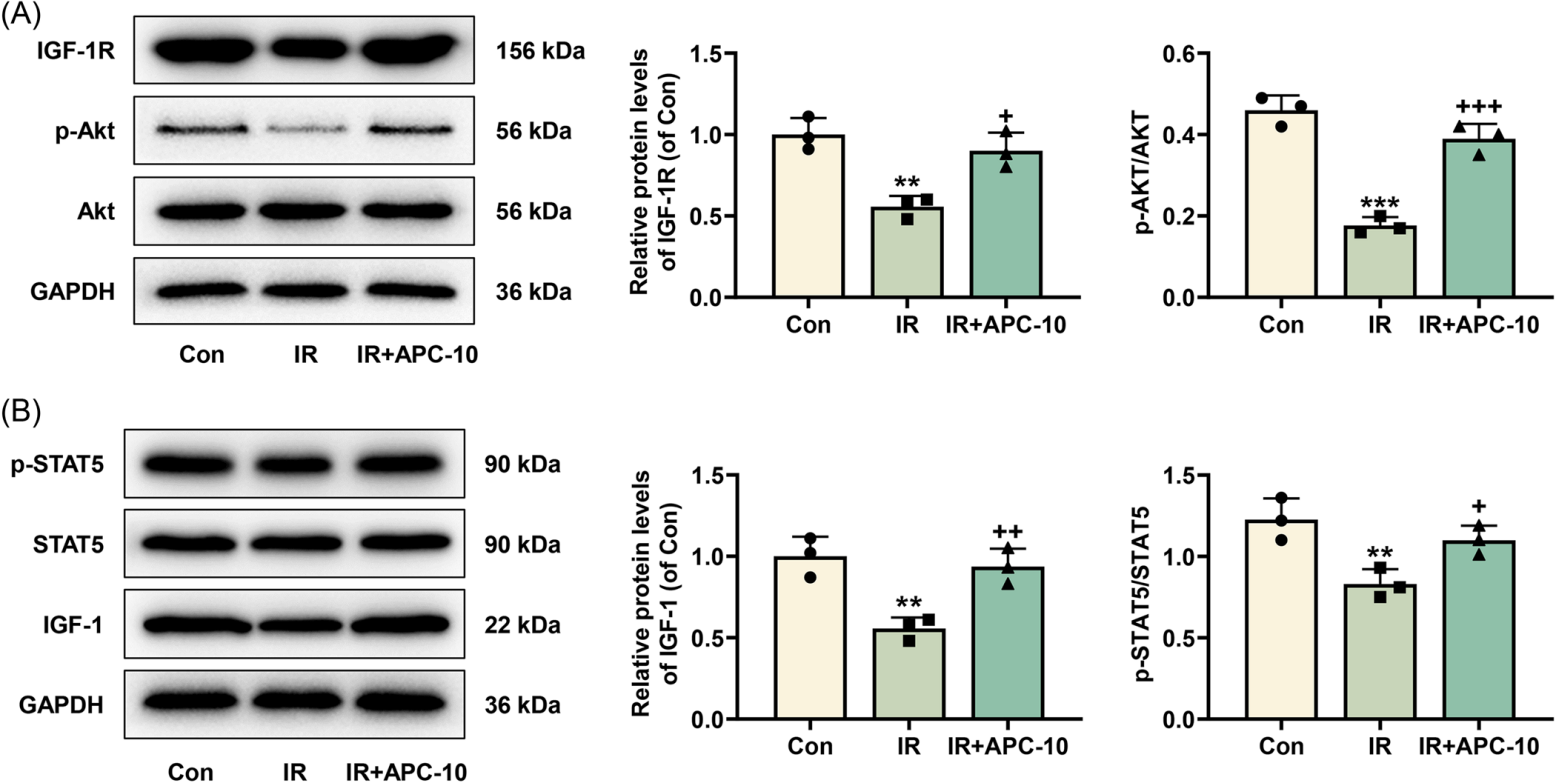
APC offset the effects of IR on the expression levels of IGF‐1R and IGF‐1 and the activation of AKT and STAT5 in HepG2 cells. (A and B) The expression levels of IGF‐1 (B), IGF‐1R (A), p‐STAT5/STAT5 (B), and p‐AKT/AKT (A) in HepG2 cells which were subjected to IR induction and then treated with 10 mg APC were analyzed by Western blot, with GAPDH serving as the control gene. ^+^ *p* < .05; **p or ^++^ *p* < .01; ***p or ^+++^ *p* < .001; * versus Con; ^+^ versus IR. AKT, protein kinase B; APC, astragalus polysaccharide; Con, control group; GAPDH, glyceraldehyde 3‐phosphate dehydrogenase; IGF‐1, insulin‐like growth factor 1; IGF‐1R, insulin‐like growth factor 1 receptor; IR, insulin resistance; p‐, phosphorylated‐; STAT5, signal transducer and activator of transcription 5.

IR can lead to reduction of AKT signaling, and STAT5 activation has been found to be detrimental to T2DM‐associated IR.^17^, ^30^ In this regard, we detected the status of STAT5 and AKT, determined the expression levels of markers that exert a hypoglycemic effect on HepG2/IR cells, and also evaluated their response to APC treatment. Through Western blot, we observed that the protein expression levels of IGF‐1R, p‐AKT/AKT, IGF‐1, and p‐STAT5/STAT5 were all decreased in HepG2/IR cells, as contrasted with those in the control cells (*p* < .01, Figure 2A,B). It was noteworthy that these decreases in the protein expression levels were offset by APC treatment (*p* < .05, Figure 2A,B).

### 3.3 AG490 reversed the impacts of APC upon the glucose intake, expression levels of IGF‐1R and IGF‐1, and the activation of AKT and STAT5 in HepG2/IR cells

**Figure 3:**
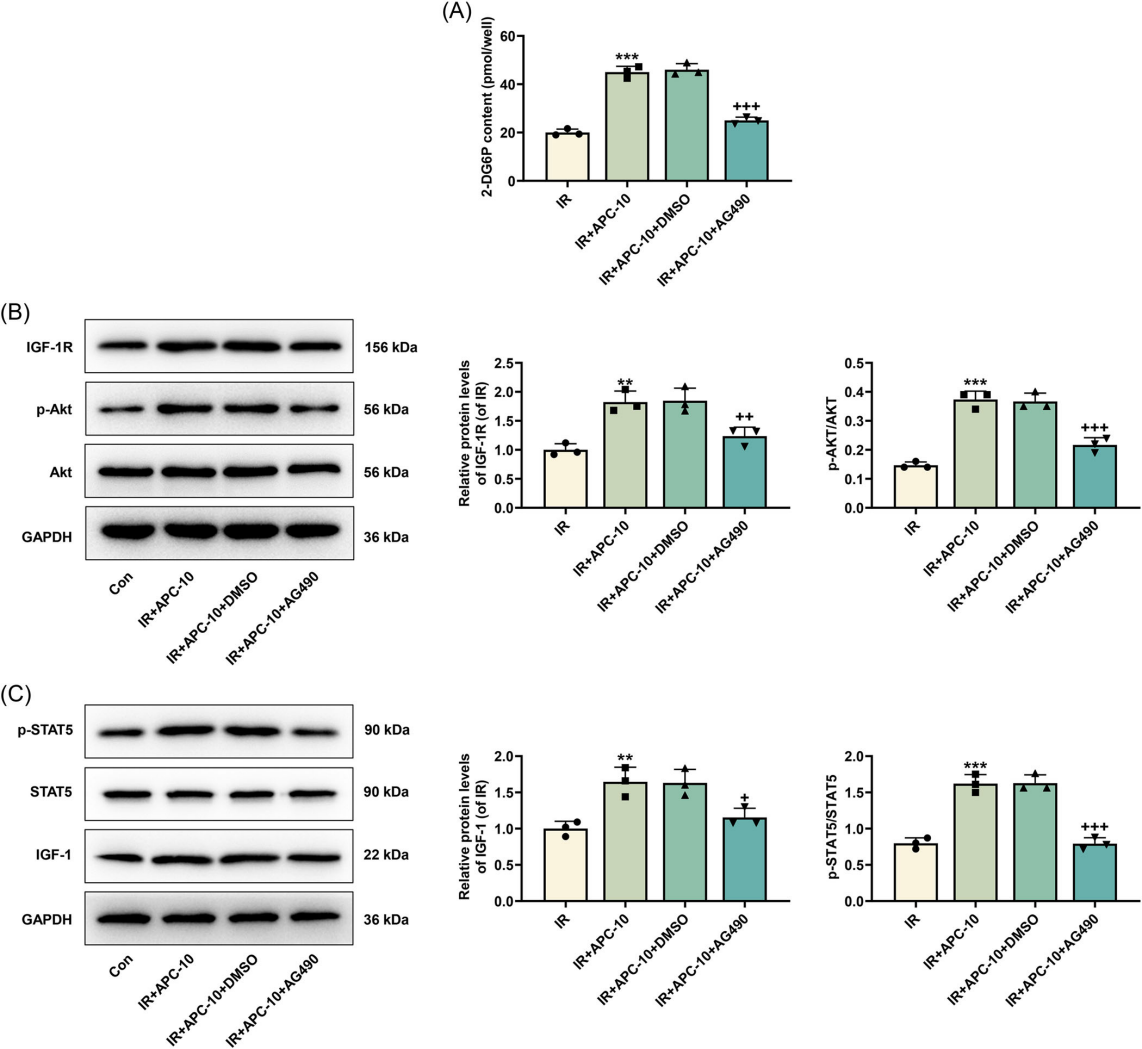
AG490 reversed the role of APC in the glucose intake, expression levels of IGF‐1R and IGF‐1, and the activation of AKT and STAT5 in HepG2/IR cells. (A) Glucose uptake level in AG490/DMSO‐pretreated HepG2 cells which were subjected to IR induction and subsequently treated with 10 mg APC was reflected by 2‐DG6P content determined through colorimetric assay. (B and C) Expression levels of IGF‐1 (C), IGF‐1R (B), p‐STAT5/STAT5 (C), and p‐AKT/AKT (B) in AG490/DMSO‐pretreated HepG2 cells which were subjected to IR induction and subsequently treated with 10 mg APC were analyzed by Western blot, with GAPDH serving as the control gene. ^+^ *p* < .05; **p or ^++^ *p* < .01; ***p or ^+++^ *p* < .001; * versus IR;^+^ versus IR + APC‐10. 2‐DG6P, 2‐deoxyglucose‐6‐phosphate; AKT, protein kinase B; APC, astragalus polysaccharide; Con, control group; DMSO, dimethyl sulfoxide; GAPDH, glyceraldehyde 3‐phosphate dehydrogenase; IGF‐1, insulin‐like growth factor 1; IGF‐1R, insulin‐like growth factor 1 receptor; IR, insulin resistance; p‐, phosphorylated‐; STAT5, signal transducer and activator of transcription 5.

AG490, an inhibitor of the JAK/STAT signaling pathway, was employed to investigate whether the actions of APC are dependent on STAT5 activation. In comparison with DMSO pretreatment, AG490 pretreatment was confirmed to reverse APC‐induced increase of 2‐DG6P content (*p* < .001, Figure 3A) and upregulation of IGF‐1R, p‐AKT/AKT, IGF‐1, and p‐STAT5/STAT5 in HepG2/IR cells (*p* < .05, Figure 3B,C).

### 3.4 AG490 counteracted the inhibitory effect of APC on IGF‐1 secretion in HepG2/IR cells

**Figure 4:**
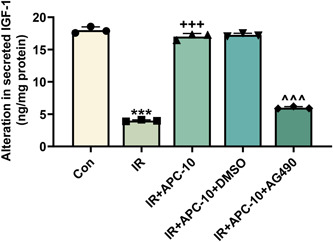
AG490 counteracted the inhibiting impact of APC on IGF‐1 secretion in HepG2/IR cells. (A) Radioimmunoassay was used to detect IGF‐1 secretion from HepG2 cells which were pretreated with AG490/DMSO, then subjected to IR induction and eventually treated with 10 mg APC. ^+++^ *p*, ***p, or ^^^^^ *p* < .001; ^+^ versus IR; * versus Con; ^^^ versus IR + APC^−^10. APC, astragalus polysaccharide; Con, control group; DMSO, dimethyl sulfoxide; IGF‐1, insulin‐like growth factor 1; IR, insulin resistance.

The alteration in the level of IGF‐1 secreted from HepG2 cells was analyzed in APC‐treated HepG2/IR cells in response to AG490 pretreatment. Through RIA, the level of secreted IGF‐1 was found to drop in HepG2/IR cells, compared to that in the control cells, which, however, was offset by APC treatment (*p* < .001, Figure 4). Notably, AG490 pretreatment further counteracted the role of APC treatment in IGF‐1 level (*p* < .001, Figure 4).

## 4 DISCUSSION

IR, a powerful risk factor for T2DM, is manifested by reduced glucose uptake resulting from the failure of insulin to stimulate glucose disposal.^2^ APC, a traditional Chinese medicine, is widely used as an antidiabetic agent.^25^ Considerable evidence has demonstrated that APC exerts a potent therapeutic effect against IR via modulating glucose metabolism to enhance glucose uptake.^26^, ^29^, ^31^ However, the underlying mechanism still needs further investigations.

The liver is a place where glucose and lipid are metabolized most frequently, thus playing a vital role in regulating insulin, glucagon, GH, epinephrine, and other hormones.^32^ This role enables the liver to participate in energy metabolism of various tissues and organs.^32^ In the liver, insulin inhibits a complex metabolic process, gluconeogenesis, thereby promoting macromolecule glycogen synthesis and suppressing hepatic glucose production.^33^ Hepatic IR can cause unrestrained gluconeogenesis, resulting in hyperglycemia.^33^ Even worse, hepatic IR also induces IR in other tissues.^33^ Therefore, hepatic IR is at the top priority to be combated. A previous study has proven that APC suppresses hepatic IR and abnormal glycolipid metabolism by improving hepatic sirtuin 1‐peroxisome proliferator‐activated receptor α‐fibroblast growth factor 21 intracellular signaling in catch‐up growth rats.^34^ Besides, APC has been discovered to downregulate the ubiquitination level of insulin receptor substrate (IRS)‐1 in the liver of IR mice to block the progression from hepatic IR to diabetes.^35^ Meanwhile, the insulin‐sensitizing potential of APC has been confirmed by a study using the HepG2/IR cell model.^36^ Here, in our study, through viability assay in HepG2/IR cell models, we found that APC at 10 and 20 mg can evidently protect HepG2/IR cells. Improved insulin sensitivity and weakened IR both lead to enhanced glucose uptake.^25^, ^26^, ^27^ 2‐DG6P is a glucose uptake metabolite, whose decreased level indicates an attenuated glucose uptake capacity^37^ that can lead to IR.^2^ Our study showed that APC was able to abrogate IR‐induced IR‐2‐DG6P content decrease, suggesting that APC suppressed hepatic IR.

IGF‐1 is a key hormone in the pathophysiology of IR.^38^ Dysregulation of its interaction with GH and insulin can lead to metabolic syndrome including IR, which increases the risk of T2DM up to fivefold.^38^ Previously, several studies have discovered that promoted synthesis of IGF‐1 in the liver facilitates glucose uptake,^39^ and progressive reduction in circulating IGF‐1 level is associated with enhanced IR, glucose intolerance, and T2DM.^40^, ^41^ IGF‐1 can act on IGF‐1R. The binding of IGF‐1 to IGF‐1R triggers the activation of the tyrosine kinase domain of IGF‐1R, and subsequent phosphorylation of members from the IRS family.^42^, ^43^ This phosphorylation initiates recruitment of downstream effectors including the p85 regulatory subunit of PI3K, and amplified PI3K signaling activates (phosphorylates) AKT, leading to augmented glucose transport, glycogen and protein synthesis.^44^, ^45^ Previous studies have presented that dietary supplementation of APC in sows can improve immune components accompanied by an increased level of IGF‐1 in their colostrum,^46^ and APC improves insulin sensitivity through activating AKT.^26^ Similarly, our study revealed that APC abrogated IR‐induced inhibition on the expressions of IGF‐1 and IGF‐1R proteins, IGF‐1 secretion as well as AKT activation, signifying that APC inhibited IR through activating the IGF‐1 pathway.

A prior research on anti‐inflammatory potential of APC has evidenced that APC‐delivered improvement of colitis in rats involves the upregulation of STAT5a.^21^ Activation of STAT5 has been found to be indispensable in NO‐1886‐induced IGF‐1 secretion and CYP7A1 upregulation that suppresses diet‐induced IR.^17^ Besides, STAT5 activation is required for the prolactin receptor‐mediated increases in hepatic insulin sensitivity.^47^ In our study, APC that launched anti‐hepatic IR actions, as evidenced by our aforementioned results, was found to activate STAT5. Subsequently, AG490, an inhibitor of the JAK/STAT signaling pathway, was employed to verify whether STAT5 activation played an indispensable role in the actions of APC on IR. Our results denoted that AG490 counteracted all the effects of APC mentioned above on the in vitro hepatic IR model.

In conclusion, the present study uses HepG2/IR models to demonstrate that APC ameliorates hepatic IR and related glycometabolism, and further confirms the necessity of STAT5 activation in the effects of APC. Since STAT5 activation is indispensable to IGF‐1 upregulation in anti‐IR activity,^17^ we suggest that APC activates the STAT5/IGF‐1 pathway, thereby combating hepatic IR. Our findings may provide a novel avenue for alleviating IR, thus providing scientific basis for the improvement of type 2 diabetes. Due to the complex mechanism of APC, there are still many other pathways involved in the mechanism of action of APC, except for the STAT5/IGF‐1 pathway. This study only reveals a small part of them, and more mechanisms will be investigated in the future. In addition, in vivo IR models are expected to be established for verifying the underlying mechanism of treatment with APC on IR.

## AUTHOR CONTRIBUTIONS

**Xinxin Yue**: Review and editing (lead); conceptualization (lead); writing—original draft (lead); formal analysis (lead); writing—review and editing (lead). **Wei Hao, Min Wang, and Yang Fu**: Software (equal); methodology (equal); writing—review and editing (supporting); conceptualization (supporting); writing—original draft (supporting).

## CONFLICT OF INTEREST STATEMENT

The authors declare no conflict of interest.

## Data Availability

The analyzed data sets generated during the study are available from the corresponding author on reasonable request.
